# Implications of national tax policy on local pharmaceutical production in a southwestern state nigeria – qualitative research for the intersection of national pharmaceutical policy on health systems development

**DOI:** 10.1186/s12913-022-07579-1

**Published:** 2022-02-28

**Authors:** Taiwo A. Obembe, Adebisi B. Adenipekun, Oyewale M. Morakinyo, Kehinde O. Odebunmi

**Affiliations:** 1 Department of Health Policy and Management, Faculty of Public Health, College of Medicine, University of Ibadan, Ibadan, Nigeria; 2 Lighthouse Global Health Initiative, Federal Capital Territory, Abuja, Nigeria; 3 Department of Environmental Health Sciences, Faculty of Science, Tshwane University of Technology, Staatsartillerie Road, Pretoria, South Africa; 4 Department of Palliative Medicine, University College Hospital, Ibadan, Nigeria

**Keywords:** Essential medicines, Local pharmaceutical manufacturers, Tax and Tariff, Price and affordability, Access to medicines

## Abstract

**Background:**

Universal access to quality and affordable medicines is one of the targets of the Sustainable Development Goals (SDGs). Access to essential medicines is defined as the availability of medicine at an affordable price in public and private health institutions including retail pharmacies in the proximity of less than an hour from the home of the population. The dependence on importation to meet the drug needs of an expanding population has implications on the growth of the local pharmaceutical industry, availability, and affordability of essential medicines in the country. This study aims to understand the dynamics of tariffs and tax policy on local drug production in the pharmaceutical industry in Nigeria.

**Methods:**

This was a qualitative study involving the use of Key Informant Interviews (KIIs). A total of 15 stakeholders were interviewed. Interviews conducted were analysed thematically. The data generated were analysed using Atlas.Ti version 8.2.

**Results:**

Assessment of the pharmaceutical industry sub-sector underscores myriads of challenges facing the industry and explains why the pharmaceutical industries in Nigeria are performing sub-optimally. Key stakeholders in the sector expressed concern about the fact that substantial percentage of drugs consumed in Nigeria are imported. Local manufacturers are underperforming due to several factors. Some highlighted factors were unfriendly tax environment, high cost of production, infrastructural deficit, low patronage from the government, and lack of access to low-interest rate loans. However, tax incentives and tax cuts are proven strategies to encourage and facilitate the growth of entrepreneurs in the pharmaceutical industry.

**Conclusions:**

Stakeholders’ perspective of implications of the tax environment on the pharmaceutical sector of Nigeria revealed the unfriendliness of the government policy to local manufacturers. Although the checklist for availability and prices of essential medicines in Osun state, Nigeria revealed that the pre-selected drugs were available in the facilities, the observed prices further affirmed the relative difficulty that is experienced by local manufacturers to compete with imported brands of the same drugs.

## Background

Universal access to quality and affordable medicines is among the targets of the third Sustainable Development Goal (SDG 3) which is focused on the health and well-being of people across the world [1]. Essential medicines, as defined by the World Health Organization (WHO), are medicines that are a priority for the healthcare and wellbeing of a population. These medicines are required to be available at all times at the right quality, quantity, condition, dosage forms, and price that is affordable for individuals, the community, and the country [2].

Access to essential medicines is defined as the availability of medicine at an affordable price in public and private health institutions including retail pharmacies nearby in less than an hour’s walk from the home of the population [3]. It has been reported that at least one-third of the global population do not have access to medicines [4]. The burden of inaccessibility of essential medicines is higher in low- and middle-income countries (LMIC) [5]. Hence, government-owned hospitals and facilities in these countries usually experience consistent stock-outs of essential medicines thus making access to medicines more difficult and expensive for their population, many of whom live below the poverty line [6–8].

The issue of access to medicines is a priority to policy-makers in both high-income and low-income countries to ensure that their healthcare system is effective in meeting the need of the population [9, 10]. This has led to government policies such as the exemption of pharmaceutical products from import duties in many developing countries [11, 12]. However, several factors influence the availability and affordability of drugs; these include the availability of local pharmaceutical production, price of the medicines from the manufacturers, added cost across the supply chain, sanity and responsiveness of the distribution channels, the income of the population, and some measure of an unacceptable burden of cost [6, 13, 14].

The concept of affordability is a normative issue, hence it is very difficult to define and operationalize [15]. However, the importance of being able to ascertain when a good or commodity (in this context, essential medicine) is too expensive for someone to procure makes a working definition for affordability non-negotiable. Some scenarios that can be used to explain the affordability of essential medicines are;

*Essential medicines can be considered not affordable if the price exceeds the total budget an individual can attract. The drawback of this model is that it disregards all other spendings that an individual needs to do*.

*Essential medicines can be considered not affordable if after the procurement the individual falls below the poverty line. The challenge with this model is the determination of the level at which the poverty line is set*.

The methods of calculating the affordability of essential drugs include impoverishment and catastrophic payment methods which are calculated regarding actual income in the population. Both methods automatically consider income distribution. The World Health Organization (WHO) and Health Action International (HAI) have developed an alternative methodology for measuring the affordability of medicines using the number of days’ wages the lowest-paid unskilled government worker (LPGW) needs to spend to procure the complete treatment course using a particular medicine [6, 16].

The local production capacity of pharmaceutical companies in Nigeria cannot be separated from the monetary and tax policies in the country. The quality and extent of infrastructural development and the ease of doing business in the country are all economic realities that influence actors in the pharmaceutical industries to either produce locally or import essential medicines. Macro-economic policies such as the revised National Tax Policy of Nigeria have the potential to strengthen or weaken the local pharmaceutical production and by extension, the availability and affordability of essential medicines in Nigeria. Currently, almost all pharmaceuticals (raw materials and finished products) are imported from countries like India and China [17].

Nigeria can be said to be experiencing a population explosion as the national population was estimated to be over 210 million as of 2020 [18]. As such, the healthcare needs of its citizens are equally increasing. However, there is still a high level of inequality in the socio-economic status of citizens with 90% of people still living below the poverty line and having very low purchasing power. In a study conducted on health expenditures using household data, 78 million people have falling below poverty line when their healthcare expenditure is removed from their incomes [19]. Findings from another study that calculated the affordability of medicine for four essential medicines across 16 Low-and-middle-income-countries (LMICs) with a total population of 775 million people showed that the medicine with the lowest cost would be unaffordable for 140 million people in these countries [20].

The heart of the healthcare system centres around the provision of essential medicines and the pharmaceutical sector is directly saddled with the responsibility of ensuring that essential medicines are available, accessible, and affordable to Nigerians [21]. In addition to meeting the needs of Nigerians, evidence from developed countries such as the United Kingdom and the United States of America show that the pharmaceutical industry can add significant value to the nation’s economy which is struggling for recovery and growth [22]. There are over 120 pharmaceutical industries in Nigeria with a higher percentage of indigenous ownership [23], accounting for about 60% of ECOWAS pharmaceutical industry capacity. Despite the vibrant indigenous pharmaceutical industry in Nigeria, 80% of drugs in Nigeria are imported [24]. The dependence on importation to meet the drug needs of an expanding population has implications on the growth of the local pharmaceutical industry, accessibility, availability, and affordability of essential medicines in the country [22–24]. This study thus sought to understand the dynamics of tariffs and tax policy of the pharmaceutical industry on local pharmaceutical production in Nigeria.

## Methods

Study design

This was a qualitative study involving the use of Key Informant Interviews (KIIs). The study population comprised key stakeholders such as Nigeria Customs Services (NCS), Federal Inland Revenue Service (FIRS), National Agency for Food and Drugs Administration and Control (NAFDAC), Pharmacists Council of Nigeria (PCN), Pharmaceutical Society of Nigeria (PSN), Pharmaceutical Manufacturing Group of Manufacturers Association of Nigeria (PMG-MAN) and National Association of Industrial Pharmacists (NAIP). 

### Study setting, participants and sampling

The interviews were conducted as face-to-face interviews or phone interviews in locations preferred and arranged by the participants as convenient for their schedules.

Osun state is one of the six south-western states in Nigeria. The state capital is Osogbo and the state is divided into three federal senatorial districts, each of which is composed of two administrative zones.

### Data collection

Inclusion criteria for this study was that the stakeholder must have been in the public or private sector in relation to taxation, duties, and pharmaceuticals for not less than 6 years of work experience. All participants that met the inclusion criteria were approached to participate in the study. A total of 21 stakeholders were approached by phone call and only 6 declined to participate in the study. Amongst those who met the inclusion criteria and that agreed to participate in the study, none declared any conflict of interest. Thus, a total of 15 stakeholders eventually participated in the key informant interviews.

Out of the 15 interviews conducted, 6 were conducted virtually through recorded phone interviews. The face-to-face interviews were recorded with a digital recorder. Both physical and virtual interviews varied in duration, but all the interviews were between 30 min to 1 h in duration.

After explaining the research to the participants in detail, informed consent was obtained to conduct and audiotape the interviews. Consent for the presence of a note-taker was also obtained and the importance of the note-taker was explained to the respondents before the commencement of every interview. The four principles of good ethics were put into consideration which includes the principles of non-maleficence, beneficence, justice, and respect for autonomy [25]. All data obtained during the study were treated with utmost confidentiality and anonymous identifiers used. In total, four people were involved in data collection and analysis (principal investigator inclusive). Data access was restricted to these four personnel; two research assistants that were responsible for the data collection, the data analyst, and the principal investigator. All data collected during this study were stored in a device that is secured with a password. The confidentiality of research participants was not compromised. Participants had their autonomy and reserved their rights to participate, decline and/or withdraw participation at any time during the entire course of this study.

All interviews were transcribed verbatim. All interviews were conducted in English.

Analysis and coding of data commenced after data collection had been completed for all participants. The transcribed data were imported into a qualitative computerized qualitative data analysis program, Atlas.Ti vs 8.2 qualitative analysis software [26]. In the software, interview texts were coded by meaning into themes with framework analysis using the deductive approach. Relationships between key issues of taxation and local pharmaceutical production in Nigeria were examined. New, but relevant emerging themes were grouped.

### Data analysis and rigour

The raw qualitative data were in the form of audio-recorded discussion and summary notes. The first step was to transcribe the recordings into a word document. Transcripts were analysed thematically. The data generated were analysed using Atlas.Ti version 8.2. This qualitative study utilised both thematic and content analysis using the deductive approach.

Thematic analysis involved the identification, analysis, and reporting of patterns in data and provided the basis for many other forms of qualitative analysis [27]. The process of thematic analysis involved careful identification of themes achieved through familiarization and immersion in data [28]. Thematic analysis was used to explore contextual situations, experiences, and perceptions integrating meanings at an individual or group level highlighting important emerging themes [29]. Content analysis, on the other hand, allowed for systematic coding and categorizing large amounts of data to explore trends, patterns, frequency, and relationships. Content analysis was a useful exploratory tool to identify common issues in the data [27]. The data management was done using Atlas.Ti Vs 8.2.

### Ethical considerations

Approval to conduct this study was obtained from the University of Ibadan /College of Medicine, Institute for Advanced Medical Research and Training (IAMRAT) UI/UCH EC Registration (NHREC/05/01/2008a). The research and ethics committee of the University of Ibadan /College of Medicine, Institute for Advanced Medical Research and Training (IAMRAT) approved this study. 

## Results

A total of 15 key actors in the pharmaceutical sector were interviewed for this study as shown in Table 1.

**Table 1:** Socio-demographic profile of respondents

S/N	AREA OF PRACTICE	ACRONYM	GENDER	YEARS OF EXPERIENCE
1	Community pharmacy	CP	Male	20 years
2	Community pharmacy	CP	Male	7 years
3	Nigeria Customs Service	NCS	Female	6 years
4	Federal Inland Revenue Service	FIRS	Male	10 years
5	Nigeria Customs Service	NCS	Male	10 years
6	Hospital pharmacy	HP	Male	9 years
7	Industrial pharmacy	IP	Male	6 years
8	Industrial pharmacy (Local manufacturer)	IP-LM1	Male	15 years
9	Industrial pharmacy (Local manufacturer II)	IP-LM2	Male	25 years
10	National Agency for Foods and Drugs Administration and Control	NAFDAC	Male	15 years
11	National Association of Industrial Pharmacists	NAIP	Male	30 years
12	Pharmacists Council of Nigeria	PCN1	Male	6 years
13	Pharmacist Council of Nigeria	PCN2	Female	10 years
14	Pharmaceutical Society of Nigeria	PSN1	Male	15 years
15	Pharmaceutical Society of Nigeria	PSN2	Male	30 years

### Perception of Stakeholders on Performance of Pharmacy practice in Nigeria

Stakeholders interviewed believed the different areas of pharmacy practice in Nigeria are performing poorly. Below are some quotations about the pharmacy sector in Nigeria;

“*Generally, it has always been said that the pharmaceutical industry in Nigeria is chaotic. The word chaotic has been in use for over 30 years and it is appalling that we still have to go with the same terminology especially when it comes to drug distribution. The outlook of pharmacy practice in Nigeria is not good enough.”* (KII 1_CP_Male)

While some of the stakeholders opined that the industry was growing at a marginal pace, others believed that the industry is just chaotic (unorganized and disorderly) and underperforming. Several factors were attributed to the performance of the industry. Below are some quotations about the pharmaceutical industry in Nigeria performing below expectations:

“*As of today, pharmaceutical manufacturing companies in Nigeria are underperforming. Not in terms of quality but in terms of what they have the potentials to produce. I don’t know any manufacturing company in Nigeria that does beyond 50% of its installed capacity. Here, we have been doing 20 to 25% of our installed capacity because of competition by government policies which support the importation of parallel products that we can produce here in the country as finished products. We are not yet seeing the need to industrialize Nigeria. Look at the statistics of local manufacturing companies in Nigeria, are they increasing? They are folding up for obvious reasons.”* (KII 9_IP-LM2_Male)

“*The pharmaceutical industry in the country is performing below average. We know that most of the companies are not producing ethical (prescriptions only medicines) products. They do mostly Over the Counter (OTCs) medicines.”* (KII 7_IP_Male)

“*The local pharmaceutical industry in Nigeria particularly the manufacturing sector has experienced some growth in the past 33 years but in terms of capacity, what we have now is just about 42 percent and that has got to do with how things are done in Nigeria. We spend too much on import, fastest way to do things, forgetting that we need to develop our local capacity.”* (KII 11_NAIP_Male)

### Importation versus local manufacturing in Nigeria

With a fast-growing population of over 200 million people, the need for a constant supply of quality drugs for Nigerians is substantial. Based on the principle of demand and supply, the drug needs of Nigerians must either be met by importation or local manufacturing. Key stakeholders in the sector expressed concern about the fact that a greater percentage of drugs consumed in Nigeria were imported.

“*The percentage of locally produced medicines is very low for now because to start with, the number of manufacturers we have in Nigeria is not much and they don’t produce every drug. As regards products like paracetamol, we can say we have a larger percentage of it being produced in Nigeria but when we talk of some other products, you realize that majority of them are imported.”* (KII 14_PSN1_Male)

“*Imported products are higher. Even in the pharmacy here, we have close to 80 to 85% of the products on the shelf as imported while the locally manufactured ones are just about 10 to 15%. Even the ones that are manufactured close to us here in Osogbo, we still have issues of costing with them. The truth is that any good business owner will prefer to import rather than set up a manufacturing plant in Nigeria.*” (KII 1_CP_Male)

Although the percentages of locally produced drugs quoted by the respondents varied, they all contributed based on their scope of knowledge and areas of professional practice in the sector. They all, however, have one thing in common – Nigeria still depends largely on the importation of drugs. These have consequences on the growth of the pharmaceutical subsector and the economy of the nation at large.

“*Definitely it has a negative impact. It is so bad that some cough syrups are being imported into the country. Cough syrup is a preparation that pharmacy students can make. This shows the extent of the negative impact of importation*.” (KII 6_HP_Male)

“*Well, importation has always been there. Just that, what is expected of us is that we are growing to reduce the impact of importation. We have grown actually but in terms of development, we have not really developed much. Many of the active ingredients needed for production are still imported. So, we have grown but have not developed*.” (KII 11_NAIP_Male)

### Effect of Importation on Availability of Essential Medicines in Nigeria

Stakeholders believed there was no significant shortage in essential medicines in Nigeria because such demand-pull creates business opportunities for drug importers.

“*Essential medicines are readily available because they are imported by the drug importers. Just that importers get richer. The capitalists know the drugs that are required and import them to meet their needs.*” (KII 2_CP_Male)

“*I want to say that the essential drugs will continue to be short. As long as we don’t see any sense in manufacturing on our own, the drugs will always be brought in as imported. There are other variables too that control how these things come in. Availability will always be hampered because either foreign exchange (FOREX) is not available for them to trade or there is port congestion as we are currently having it in Nigeria. Some will be delayed for whatever reasons such that when drugs are needed, it may be short and scarce. Of course, the price will skyrocket.”* (KII 9_IP-LM2_Male)

### Effect of Taxation on Local Production of Drugs in Nigeria

Interviewed stakeholders shared their experiences and perception of the tax environment in Nigeria and how it affects local pharmaceutical growth.

Some of the emerging sentiments from the interviews with the stakeholders were that taxes and tariffs are major avenues to generate revenue by the government to keep an economy up and running. It is also a civic responsibility for individuals and companies to pay tax to the government. However, tax cuts and tax breaks are proven strategies to encourage entrepreneurs, an exercise geared at facilitating growth in certain sectors of the economy. The following quotes buttressed the points expressed by the stakeholders.

*For local industries, pharmaceutical products are exempted from VATs because it has to do with health and every human being, rich and poor access health facilities. Medical facilities have to be affordable and accessible. Adding VATs to it makes it more expensive and you know that based on the concept of VATs, it is the final consumer that bears the cost. So, in order to encourage local growth to boost our GDP, we have encouraged local manufacturers and exempted pharmaceuticals from VATs.* (KII 4_FIRS_Male)

“*When you have an industry and you are contending with three tiers of government to pay tax, how do you survive? Look at the state of the economy. Even if you want to pay your tax cheerfully as it is expected of a citizen, the economy is not encouraging. Different people and agencies from the three tiers of government come for tax and levies. These are the issues. The problem may not be tax alone but it also a key factor*.” (KII 7_IP_Male)

“*So up till today, they still charge us 20% on empty gelatine capsules which is ridiculous. But if you import a finished capsule product, it used to be zero percent. It is of recent that they reviewed that and raised it to 2-5% thereabouts. So how can a manufacturer import component at 15-20% and is expected to compete with the importers of finished products at 5%?*” (KII 9_IP-LM2_Male)

“*Well, the tax regime has blocked people from playing in the industry. One thing that we have is multiple taxations. Multiple taxations is an issue that was very bad in those days. You cannot even move your company vehicle from one place to another without the local government people stopping you. On the road, you have the police, customs, road safety, and other regulatory agencies. This is where multiple taxations come in when you settle one. Also, there are these ECOWAS external tariffs that remove duties on pharmaceuticals. But somehow, they have introduced another called Income Adjustment Tax, if you find yourself earning so much money. The importers and manufacturers will add it to their production cost. It is the end-users that will bear the cost and suffer the consequences.*” (KII 11_NAIP_Male)

### Effect of Revision of National Tax Policy on Local Production of Drugs in Nigeria

Participants believed there was no difference in the impact of the revision of the 2012 National Tax Policy in the year 2017. The quotations below show the position of the respondents on the effect of the revision of the National Tax Policy.

“*Well, from the line of products we are engaging with here, there isn’t any impact or difference, but I know that those who have alcohol content in their products might have been affected by such policies.*” (KII 9_IP-LM2_Male).

*The main issue with the previous tax policies is the enabling environment for implementation. Even if you dust the previous tax policies and start implementing them, you will get the desired results. Implementation has always been an issue. However, I’m not being political. Implementation is happening now compared to how it was in the past. You will have to implement something before you can identify the flaws and know whether we are ripe for it or not. If it is not implemented, it will just be theoretical rather than practical*.” (KII 4_FIRS_Male)

### Tariff on Importation of Finished Products Versus Active Pharmaceutical Ingredients in Nigeria

The participants opined differently on the tariff on the importation of finished products compared to the active ingredients in Nigeria. The customs believed that the tariff on unfinished products should be lower compared to finish products with a view to encourage local production as guided by the Nigeria Tax Policy and custom books. However, the industrial pharmacists think that making the tariff of raw materials lower than finished products adversely affects local production and encourages importation of finished products. See below some quotations to buttress these point.

“*Well, unfinished and finished don’t have the same tariff generally. Unfinished goods carry a lower tariff. For instance, in vehicle importation, some come here to assemble, and it is cheaper because, at the end of the day, they still employ people. This applies to the pharma sector too so they cannot be paying the same tariff.*” (KII 3_NCS_Female)

“*Customs have their books and they know what tax is exempt and what tax is not. Importers will have to pay duties; in your duties, they will not calculate VAT if it is VATs exempted. Fortunately, everything is now automated, so it is easier to calculate the charges and it is the system that will identify what is VATs exempted or not*.” (KII 3_NCS_Female)

“ *You will be surprised that some of the things we classify as starting materials, custom classifies it as packaging materials. For instance, gelatine capsules. We see gelatine capsules as starting material because you swallow with the API, but they insist that it is packaging materials. I do not know who swallows packaging materials with medicines. So up till today, they still charge us 20% on empty gelatin capsules which is ridiculous. But if you import a finished capsule product, it used to be zero percent. It is of recent that they reviewed that and raised it to 2-5% thereabouts. So how can a manufacturer who imports component at 15-20% compete with the importers of finished products at 5%? So, the system is encouraging trading, except if you have this patriotic heart to just build Nigeria into an industrialized world, create a job for the populace, add value, and pay tax to the government otherwise, it is not encouraging at all.*” (KII 9_IP-LM2)

## Discussion

### Factors affecting access to essential medicines

Many factors contribute to access (availability and affordability) to essential medicines. These include the local production capacity of pharmaceutical companies to meet domestic demands of medicines, tariffs and duties on imported medicines, supply chain and drug distribution system, government budget and expenditure on health, etc. [4, 6]. The key informant interviews showed that the local production of medicines in Nigeria is significantly less than the estimated drug needs of the population of the country. This finding is consistent with the report of Mohiuddin AK (2019) on the experience of Bangladeshi pharma industry that had 75% of its market dominated by imported medicines until the formulation of the nation’s National Drug Policy (NDP) which led to a dramatic positive changes for the country’s local pharmaceutical industry. The report showed tremendous growth in Bangladesh’s pharma sector but also some challenges facing the country’s pharma sector. Some of the challenges include economic development, population blast, and investment scopes [30].

Challenges such as population explosion and macro-economic conditions which drive economic policies including national tax and tariff decisions are not peculiar to Nigeria. In Nigeria, macroeconomic policy is usually implemented through two sets of tools which are fiscal and monetary policy. The impact aim of the macro-economic policy is to stabilize the economy. However, rather than depend on the importation of medicines, countries such as Iran now record upward trends in their local production capacity. As reported by Cheraghali AM (2017), the promotion of the national pharmaceutical industry is one of the main goals of Iran’s NDP. As a result, the share of the national pharmaceutical industry in the local pharmaceutical market has grown to 60%. Iran currently has one of the largest capacities of the production of generic medicines in the Middle East and MENA region [31].

Pakistan is a lower-and-middle-income country like Nigeria and has a double burden of communicable and non-communicable diseases and poor health indices. The country also has in common with Nigeria's reduced health sector investment, poor coverage of health insurance, absence of a strong pharmacovigilance system, and challenges with counterfeit medications. However, the country has a vibrant pharmaceutical sector fulfilling 70% of the local medicine demands and producing finished pharmaceutical products considered acceptable by countries across Asia, Africa, and the United States [32]

Effect of local manufacturing of drugs on price of essential medicines.

One of the drivers of prices of essential medicines and local drug manufacturers’ growth in Nigeria is the dynamics of tariffs on unfinished products and finished products imported into Nigeria. Due to the sub-optimal performance of the petrochemical industry in Nigeria, to the point that it cannot adequately provide raw materials needed for manufacturing in Nigeria, local manufacturers still have to import Active Pharmaceutical Ingredients (APIs) for manufacturing. However, drug importers into Nigeria on the other hand manufacture their products in countries like India and China and import the finished product into Nigeria for distribution and marketing. If the tariff of the importation of finished products is significantly lower than that of those importing APIs and excipients, then the tax regime is invariably inhibiting local pharmaceutical growth.

According to the informants, local manufacturers are confronting myriads of challenges that limit the extent to which they can contribute to the level of essential medicines available in Nigeria. Some of the challenges highlighted include a very high cost of production. The high cost of production makes it difficult for local manufacturers to compete favourably with drug importers. In some cases, as observed in the prices of selected medicines surveyed with the checklist, prices of some locally manufactured drugs are equal or higher than those imported at the retail level where final consumers procure. If the cost of production for local manufacturers can reduce, there will be a reduction in the price of their products, and that price dynamics will help them be at an advantage for competition with importers thereby achieving a reduction in the price of essential medicines in Nigeria [33, 34].

Factors contributing to the high cost of production include infrastructural deficit and epileptic state of power supply. The companies are forced to run on generators and/or other alternative sources of power. Currently, almost all pharmaceuticals (raw materials and finished products) are imported from countries like India and China [17]. In these countries, infrastructural deficits that are experienced in Nigeria do not exist. Bridging the infrastructural gap, especially that of electricity will accelerate progress towards meeting the drug needs of Nigerians. Besides, evidence from developed countries such as the United Kingdom and the United States of America shows that the pharmaceutical industry can add significant value to the nation’s economy which is struggling for recovery and growth [22].

National tax policy and local pharmaceutical production in Nigeria.

The tax environment for the pharmaceutical sector is not friendly. It was found during the interviews that pharmaceutical products are Value Added Tax (VAT) exempt. However, drug importers and local manufacturers pay huge customs duties for importing Active Pharmaceutical Ingredients (APIs), excipients, packaging materials, and finished products. All these add to the cost of the product at the retail level. Apart from customs duties, local manufacturers and stakeholders in the sector also emphasized the challenges of multiple taxations and an inefficient tax system where tax dues are arbitrary.

The 2017 National Tax Policy attempted to simplify and summarize the 2012 National Tax Policy document. It is expected that the new policy document will improve the efficiency of the taxation system in Nigeria. Some of the stakeholders interviewed believe the 2017 policy has improved the taxation system in Nigeria and has led to more IGR for the government. However, others stated categorically that they have not observed any improvement in the taxation system over the years. Another important theme observed was the fact that local pharmaceutical industries are not currently enjoying any tax incentives which could help boost the sector.

It is important to note that Nigeria is a member of the World Trade Organization (WTO) and consequently a signatory to the Trade-Related Aspects of Intellectual Property Rights (TRIPS). TRIPS agreement is the first multilateral agreement to include intellectual property law into the multilateral trading system and remain the most relevant and robust multilateral agreement on intellectual property to date [26, 35, 36]. Furthermore, because TRIPS is a compulsory requirement to join the World Trade Organization, countries interested in benefiting from WTO open international markets must be ready to enact the strict intellectual property laws mandated by TRIPS. TRIPS agreement was intended to stimulate industrial growth and by extension, access to essential medicines through increased innovation and production capacity of the pharmaceutical industry.

A notable limitation of this research is the possibility of interviewer bias by stakeholders interviewed on the impact of previous tax policy on the pharmaceutical sector. Confidence to truthfully respond to questions was ensured by thoroughly explaining the research objectives to the participants. Despite this limitation, this study establishes major linkages between tax policy on the availability and accessibility of essential medicines in a low-and-middle-income sub-Saharan African country. Lessons from this study can be applied to other sub-Saharan and low-and-middle-income countries (LMIC) in the same category thus establishing its external validity.

## Conclusion

The stakeholders’ perspective of implications of the tax environment on the pharmaceutical sector of Nigeria shows that the government policy is not in favour of local manufacturers. Local manufacturers are underperforming due to several factors. Some highlighted factors in addition to an unfriendly tax environment were the high cost of production, infrastructural deficit, low patronage from the government, and lack of access to low-interest rate loans. However, tax incentives are proven strategies to encourage entrepreneurs and facilitate growth in certain sectors of the economy. Considering the aforementioned, it will be expedient to subject the tariffs on the importation of Active Pharmaceutical Ingredients and other packaging materials to a thorough review. The petrochemical industry in the country should be strengthened to serve as a source of raw materials for local pharmaceutical manufacturers. Tax incentives in form of tax breaks to local pharmaceutical companies are also recommended to encourage stimulation of growth in the industry. Furthermore, local pharmaceutical growth should remain in perspective while implementing international treaties such as TRIPS Agreement and DOHA declaration. The National Drug Policy should be implemented religiously with deliberate attention to components that require the government’s patronage of local manufacturers and enhance the manufacturer’s production capacities through single digits loans, access to financial capital, and infrastructural development such as stable electricity.

## Data Availability

The datasets used and/or analysed during the current study are available from the corresponding author on reasonable request.

## Abbreviations

CAC: Corporate Affairs Commission
CP: Community pharmacy
DOHA: Doha declaration on the TRIPS agreement and Public Health
FIRS: Federal Inland Revenue Service
FIRS: Federal Inland Revenue Services
HAI: Health Action International
HP: Hospital Pharmacy
IAMRAT: Institute for Advanced Medical Research and Training
IP: Industrial Pharmacy
KII: Key Informant Interview
LMIC: Low-and-Middle-Income Countries
LPGW: Lowest Paid unskilled Government Worker
NAFDAC: National Agency for Food and Drugs Administration and Control
NAIP: National Association of Industrial Pharmacists
NAIP: National Association of Industrial Pharmacists
NCS: Nigeria Customs Service
NCS: Nigeria Customs Services
NDP: National Drug Policy
NHREC: National Health Research Ethics Committee
OOP: Out-of-pocket payments
OTCs: Over the counter medicines
PCN: Pharmacists Council of Nigeria
PMG-MAN: Pharmaceutical Manufacturing Group of Manufacturing Association of Nigeria
PSN: Pharmaceutical Society of Nigeria
SDG: Sustainable Development Goals
TRIPS: Trade-Related Aspects of Intellectual Property Rights
UCH: University College Hospital
UI: University of Ibadan
WHO: World Health Organization
