# Correction to: Implications of national tax policy on local pharmaceutical production in a southwestern state nigeria – qualitative research for the intersection of national pharmaceutical policy on health systems development

**DOI:** 10.1186/s12913-022-07724-w

**Published:** 2022-03-10

**Authors:** Taiwo A. Obembe, Adebisi B. Adenipekun, Oyewale M. Morakinyo, Kehinde O. Odebunmi

**Affiliations:** 1 Department of Health Policy and Management, Faculty of Public Health, College of Medicine, University of Ibadan, Ibadan, Nigeria; 2 Lighthouse Global Health Initiative, Federal Capital Territory, Abuja, Nigeria; 3 Department of Environmental Health Sciences, Faculty of Science, Tshwane University of Technology, Staatsartillerie Road, Pretoria, South Africa; 4 Department of Palliative Medicine, University College Hospital, Ibadan, Nigeria

**Correction to: BMC Health Serv Res 22, 264 (2022)**

**https://doi.org/10.1186/s12913-022-07579-1**

Following publication of the original article [1], the authors identified an error in the author name of Oyewale M. Morakinyo.

The incorrect author name is: Oyewale M Oyewale.

The correct author name is: Oyewale M. Morakinyo.

The author group has been updated above and the original article [1] has been corrected.
